# Examining Delayed Recall in Cochlear Implant Users Using the Montreal Cognitive Assessment, California Verbal Learning Test, Third Edition, and Item Specific Deficit Approach: Preliminary Results

**DOI:** 10.3389/fpsyg.2021.749045

**Published:** 2021-11-04

**Authors:** Nadav Brumer, Elizabeth Elkins, Jennifer Parada, Jake Hillyer, Alexandra Parbery-Clark

**Affiliations:** ^1^Auditory Research Laboratory, Center for Hearing and Skull Base Surgery, Swedish Neuroscience Institute, Seattle, WA, United States; ^2^Department of Psychology, Bellevue College, Bellevue, WA, United States; ^3^College of Medicine, University of Arizona, Phoenix, AZ, United States

**Keywords:** cochlear implant, delayed recall, hearing loss, encoding, consolidation, retrieval, Montreal Cognitive Assessment, California Verbal Learning Test

## Abstract

**Purpose:** Recent studies using the Montreal Cognitive Assessment (MoCA) suggest delayed recall is challenging for cochlear implant (CI) users. To better understand the underlying processes associated with delayed recall in CI users, we administered the MoCA and the California Verbal Learning Test, Third Edition (CVLT-3), which provides a more comprehensive assessment of delayed recall ability.

**Methods:** The MoCA and CVLT-3 were administered to 18 high-performing CI users. For the CVLT-3, both the traditional scoring and a newer scoring method, the Item-Specific Deficit Approach (ISDA), were employed.

**Results:** The original MoCA score and MoCA delayed recall subtest score did not relate to performance on any CVLT-3 measures regardless of scoring metric applied (i.e., traditional or ISDA). Encoding performance for both the CVLT-3 and ISDA were related. Consolidation, which is only distinctly defined by the ISDA, related to CVLT-3 cued delay recall performance but not free delay recall performance. Lastly, ISDA retrieval only related to CVLT-3 measures when modified.

**Conclusion:** Performance on the MoCA and CVLT-3 in a high performing CI patient population were not related. We demonstrate that the ISDA can be successfully applied to CI users for the quantification and characterization of delayed recall ability; however, future work addressing lower performing CI users, and comparing to normal hearing controls is needed to determine the extent of potential translational applications. Our work also indicates that a modified ISDA retrieval score may be beneficial for evaluating CI users although additional work addressing the clinical relevance of this is still needed.

## Introduction

Hearing Loss (HL) and dementia are two of the most prevalent health concerns for the aging population (Kravitz et al., 2012; Olusanya et al., 2014; Rigters et al., 2018; Ogawa et al., 2019). Approximately two-thirds of adults in the United States over the age of 70 have HL and this number is expected to nearly double in the next four decades (Goman et al., 2017). Additionally, an estimated 5.8 million people in the United States and 10% of individuals 65 or older are impacted by Alzheimer’s disease, the most common type of dementia (Zhao, 2020). Mild cognitive impairment (MCI) is a distinct clinical term describing cognitive decline that can precede the formal diagnosis of dementia and is characterized by cognitive deficits not explained by typical aging (Eshkoor et al., 2015). These deficits include difficulties with memory, language, and problem-solving, without the disruption of daily living activities (Petersen, 2011). MCI, Alzheimer’s disease, and other types of dementia are commonly diagnosed by measuring performance on delayed recall tasks, among other cognitive markers (Klages et al., 2005; Dubois et al., 2014; García-Herranz et al., 2016).

**FIGURE 1:**
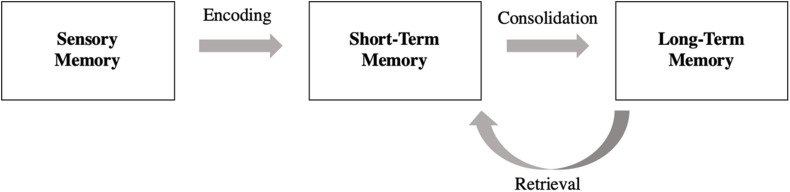
Multimodal memory model and associated cognitive processes: the sequential model begins with sensory memory and ends with long-term memory. Stimuli move through these three memory storage units through cognitive processes known as: encoding, consolidation, and retrieval.

Delayed recall is a complex skill involving multiple memory systems. Memory is believed to consist of three storage systems: sensory, short-term memory (STM), and long-term memory (LTM; Murdock, 1967; Atkinson and Shiffrin, 1968). Stimuli move through these systems via three sequential cognitive processes: encoding, consolidation, and retrieval (see Figure 1; Melton, 1963; Brown and Craik, 2000; Baddeley, 2002). Encoding refers to a mental representation or an external perceptual or sensory stimulus in the brain (Tromp et al., 2015). The stimulus is then consolidated when it is actively stored in STM, where, if it remains long enough, will be transferred into LTM, which is understood to have a capacity limited only by its ability to be accessed (i.e., retrieval; Tulving and Pearlstone, 1966). Due to the sequential nature of these processes, stimuli retrieved from LTM (e.g., delayed recall) must be encoded and consolidated first. Given the multiple cognitive processes involved, it is complicated to identify where breakdowns associated with poor delayed recall occur. For example, if stimuli are correctly recalled shortly after presentation, it can be posited that some level of encoding has occurred. Alternately, if the same stimuli are not recalled after a delay period, an impaired consolidation or retrieval mechanism is more likely to be at fault. As such, delayed recall measures are often thought to reflect retrieval abilities, whereas immediate recall tasks are meant to reflect encoding abilities (Delis et al., 2017).

Delayed recall tasks appear to present a greater challenge for individuals with HL compared to individuals without HL (Boxtel et al., 2000; Dupuis et al., 2015; Chandramouli et al., 2019). Specifically, performance on the Montreal Cognitive Assessment (MoCA; Nasreddine et al., 2005), a test used to assess cognitive functioning and screen for MCI and dementia, demonstrated that individuals with HL struggled to recall delayed recall stimuli more frequently than individuals without HL (Dupuis et al., 2015). This effect is further supported by the association between poorer baseline hearing in both ears and greater declines in delayed verbal memory found over a 2-year period (Armstrong et al., 2020). In fact, Deal et al. (2015) demonstrated an association between HL and a greater longitudinal decline in delayed recall performance over a 20-year period. Recent work suggests that using MoCA alternate scores (i.e., a scoring method whereby specific auditory subtests are systematically removed; Dupuis et al., 2015) may have clinical significance for the hearing-impaired population (Al-Yawer et al., 2019). Our own research has demonstrated that individuals with HL, specifically those with cochlear implants (CIs; surgical implants effective for those with profound hearing loss in which other assistive hearing devices are not appropriate) performed better on the MoCA presented in both a visual and auditory format when delayed recall was removed (Hillyer et al., 2020; Parada et al., 2020). However, removing test items from the total score may also decrease sensitivity (Dupuis et al., 2015). Using the California Verbal Learning Test—Third Edition (CVLT-3; Delis et al., 2017), a neuropsychological assessment of verbal learning and delayed recall, Pisoni et al. (2018) demonstrated more retrieval-induced forgetting of stimuli in delayed recall tasks in experienced CI users compared to those without HL. Additionally, CI users benefited more from semantically cued words than individuals without HL suggesting that semantic cueing allowed individuals with HL to access words that were encoded but not accessible to non-cued retrieval (Chandramouli et al., 2019; Kronenberger and Pisoni, 2019). Taken together, the current literature suggest that an impaired retrieval mechanism may underlie delayed recall deficits in individuals with HL.

While the CVLT-3 offers a more comprehensive assessment of delayed recall than the MoCA (e.g., allows for the distinction of short and long delay free and cued recall), it does not provide distinct measures of individual memory processes (i.e., encoding, consolidation and retrieval). Where traditional metrics of the CVLT-3 (e.g., learning slope, recognition-hits) reflect an overlap between memory processes (Delis et al., 1991), the Item-Specific Deficit Approach (ISDA, Wright et al., 2009) was developed with the goal of providing more distinct indices of encoding, consolidation and retrieval. The ISDA is a scoring method that evaluates list-learning performance at the item level rather than by overall trial performance across immediate recall and subsequent delayed recall trials. For example, the CVLT-3 calculates scores as a summation of total words recalled within each trial whereas the ISDA takes into account the amount of times each word has been recalled across multiple trials. This item-level approach also aims to compensate for the effects of inattention, which may prevent a participant from initially encoding a target word for later recall. This scoring method may be similarly helpful for participants with HL who may not encode a target word due to mishearing or not hearing, thus affecting their overall performance.

The aim of this study was to further explore our previous findings from the MoCA where the largest change in passing rate was observed by removing the delayed recall subtest, suggesting that delayed recall is more challenging for this patient population (Parada et al., 2020). Given the potential clinical utility of the alternate MoCA scoring methods for people with HL, we considered original and alternate MoCA scores in relation to a more comprehensive delayed recall test: the CVLT-3. As such, we administered both the MoCA as well as the CVLT-3 to gain a clearer understanding of the underlying memory processes associated with delayed recall. Given that delayed recall is a complex cognitive process consisting of encoding, consolidation, and retrieval, we also used CVLT-3 scores to produce individual ISDA indices reflective of these processes. We predicted that higher delayed recall scores on the MoCA would relate to better performance on the CVLT-3. Given that the CVLT-3 and ISDA utilize the same raw scores, we also expected that respective measures of encoding and retrieval would relate to equivalent ISDA deficit indices.

## Materials and Methods

### Participants

Eighteen (11 female, 7 male) experienced, high-performing CI users (>6 months listening experience, *M* = 56.89 months, *SD* = 34.37 months, range of 10–145 months; see Table 1 for participant CI details), between the ages of 52 and 83 years (*M* = 68.56, *SD* = 10.37) were recruited from the patient pool at the Center for Hearing and Skull Base Surgery at The Swedish Neuroscience Institute in Seattle, Washington. Experienced CI users were recruited because maximum comfortable levels and threshold levels are optimally achieved after 6 months of use and programming (Gajadeera et al., 2017). CI assisted threshold levels were not related to age (all *r* ≤ 0.282, *p* ≤ 0.929). CI users in this study were considered high-performing based on their AzBio Sentence Test (Spahr et al., 2012) percentage scores, which reflect speech perception abilities in quiet and were all above 80% (*M* = 92.78, *SD* = 6.57, range = 20%; Hillyer et al., 2020). Inclusion criteria required participants to have no recorded symptoms or diagnosis of dementia, no report of cognitive decline and no history of congenital or pre-lingual hearing loss. All participants were native speakers of English, had at least a high school education and demonstrated normal IQ scores (*M* = 107.39, *SD* = 7.96), as measured by the Test of Non-verbal Intelligence—4th Edition (TONI-4; Brown et al., 2010). All participants had a passing score for at least one of the four scoring versions of the MoCA (see Table 2 for descriptions of scoring methods). All testing procedures were approved by the Swedish Medical Center Institutional Review Board (#SWD56152-14) and participants provided informed written consent. All testing was conducted in a clinic room at the Swedish Neuroscience Institute in Seattle, WA, United States. All testing (i.e., task order and test versions) was randomized across subjects.

**TABLE 1:** Participant cochlear implant details.

**Manufacturer**	**Duration of HL (Mo.)**	**Etiology**
Cochlear Americas	40	Sudden hearing loss, acoustic neuroma
Cochlear Americas	54	Potentially genetic
Med-El	97	Unknown
Cochlear Americas	120	Potentially genetic
Advanced Bionics	120	Meniere’s disease
Cochlear Americas	144	Sudden hearing loss
Med-El	156	Potentially genetic
Med-El	167	Unknown
Advanced Bionics	211	Unknown
Cochlear Americas	222	Unknown
Cochlear Americas	254	Potentially genetic
Cochlear Americas	334	Meniere’s disease
Cochlear Americas	392	Potentially genetic
Cochlear Americas	420	Noise exposure
Cochlear Americas	480	Potentially genetic
Cochlear Americas	480	Noise exposure, potentially genetic
Med-El	534	Unknown
Cochlear Americas	636	Unknown

**TABLE 2:** Description of the standard MoCA and point allotment for original and alternate scoring methods.

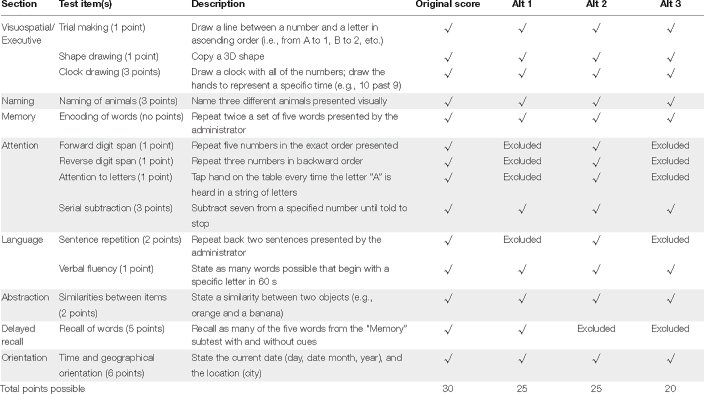

*Alt, alternate score.*

### Original Montreal Cognitive Assessment

The MoCA is a 30-point, 12-item auditory-visual neurocognitive test with eight subtests: visuospatial executive functioning, naming, memory, attention, language, abstraction, delayed recall and orientation, in that order (see Table 2 for descriptions of subtests). To evaluate delayed recall, the same five words presented in the memory subtest are recalled again after a roughly 5-min delay. Each participant was randomly assigned one of three MoCA versions (i.e., 7.1, 7.2, and 7.3) which have been shown to demonstrate equivalent reliability and validity (Costa et al., 2012; Nasreddine and Patel, 2016). In addition to standard scoring, three alternative scoring methods developed by Dupuis et al. (2015) and previously applied to CI users (Parada et al., 2020) were employed. Alternative scoring methods removed items from the attention, language, and delayed recall sections providing a means to examine the influence of specific subtests on general performance (see Table 2 for scoring methods, point allocation and cutoff scores). All MoCA subtest stimuli were administered in adherence with the MoCA test administration protocol, in either the auditory (e.g., visuospatial/executive, naming) or auditory-visual (e.g., memory, attention, language, abstraction, delayed recall, and orientation) modality. Higher scores indicated better performance.

### California Verbal Learning Test, Third Edition

The CVLT-3 (Delis et al., 2017) is a neuropsychological assessment of verbal learning and memory presented in the auditory modality. This tool aims to connect performance on subtests to specific memory deficits and strategies. Each participant was administered the CVLT-3, where 16 target words were presented and then recalled repeatedly for five immediate recall trials. Following the immediate recall trials, a distractor list of 16 non-target words was presented and recalled, after which participants were instructed to recall the original target words without, and then with semantic cues (i.e., short-delay free and short-delay cued subtests). Following a 20-min delay period, the 16 target words were recalled again without, and then with semantic cues (i.e., long-delay free and long-delay cued subtests). Lastly, the recognition-hits subtest required participants to indicate a yes or no recognition of the 16 target words amongst 32 other distractor words. The CVLT-3 also provided a calculation for learning slope, or the rate at which learning occurred during the five immediate recall trials (see Table 3 for description of all subtests and metrics). Higher scores indicated better performance.

**TABLE 3:** CVLT-3 metrics.

**Measure**	**Definition and calculation**
**California Verbal Learning Test 3**	
List A: target words	Target list (16 words), presented 5 times for trials 1–5
List B: distractor words	Interference list (16 words), presented once after list A trials 1–5
Immediate free recall (IFR): Trials 1–5^E/C^	List A is read aloud, and the participant is asked to recall as many words as possible; this is completed 5 times for a total of five trials (trials 1–5).
Learning slope^E^	The rate of learning calculated by the number of new words learned on each immediate free recall trial (i.e., difference between trial 1 and 2, trial 2 and 3 etc.).
Short delay free recall (SDFR)^R^	Number of words recalled from List A, after listening to and recalling words from List B (interference list).
Short-delay cued recall (SDCR)^R^	Number of words recalled from List A. Participants are provided verbal cues using semantic categories related to words from List A.
Long-delay free recall (LDFR)^R^	Number of words recalled from List A after a 20 min delay.
Long-delay cued recall (LDCR)^R^	Number of words recalled from List A after a 20 min delay. Participants are again provided verbal cues using semantic categories related to words from List A.
Recognition-hits^R^	Number of correctly identified target words from List A, indicated with a “yes” or a “no,” in a list of 48 presented words (32 distractor words).

*E, encoding measure; C, consolidation measure; R, retrieval measure.*

### Item-Specific Deficit Approach

The ISDA (Wright et al., 2009) is a scoring method that can be applied to any episodic memory test with multiple learning trials. The ISDA has been shown to have strong internal consistency for descriptive scales comprised of a small number of items (58–77%; Pedhazur and Schmelkin, 1991; Kehoe, 1994; Kline, 2005) and demonstrates an advantage over other traditional indices for predicting low memory performance (Wright et al., 2009). Compared to standard scoring methods, the ISDA weighs delayed recall performance more heavily in order to reflect one’s multimodal memory processing abilities. The ISDA scoring method was applied to CVLT-3 raw performance scores to calculate indices of encoding, consolidation, and retrieval (see Table 4). These indices reflect multimodal processes associated with delayed recall (see Figure 1) and therefore offer additional insight into delayed recall abilities not specifically isolated in the MoCA or CVLT-3. Because the ISDA requires participants to recall a target word during all four delayed recall subtests to avoid receiving a point toward a poorer overall retrieval deficit score, we also created an alternate ISDA retrieval index scoring method with a less stringent criteria. This alternate method requires the participant to recall a target word during at least three of the four delayed recall subtests, allowing the participant to miss the target word once before increasing their measured retrieval deficit, while also ensuring the target word is recalled on at least one short and long delay subtest. ISDA scores were calculated as a deficit, so higher scores reflected lower performance.

**TABLE 4:** ISDA metrics: for all measures, a higher score indicates a greater deficit and hence lower performance.

**Item specific deficit approach (ISDA)**	
ISDA encoding index (E)	The n of items from List A (target list: 16 words) that were recalled 2 or fewer times during trials 1–5.
ISDA consolidation index (C)	The sum of individual items that were recalled during trials 1–5, but not recalled on any delayed recall trial.
ISDA retrieval index (R)	The sum of the individual items that were recalled during trials 1–5, but inconsistently recalled across delayed recall trials.

### Statistical Analyses

Statistical analyses were completed using SPSS Version 22 (IBM Corp, 2017). Prior to analysis, normality of data was evaluated using Shapiro-Wilk tests. All measures were confirmed to be normally distributed with the exception of performance on the MoCA delayed recall subtests, and alternative 2 and 3 scoring methods. Furthermore, CVLT-3 data were analyzed relative to the mean normative scores provided by Delis et al., 2017 and confirmed to be within the normative range (i.e., T-scores between 30 and 70). While the ISDA does not have normative ranges, all data points were confirmed to be within 2 standard deviations of the mean. Age was significantly related to all CVLT-3 (all *r* ≤ −0.797, *p* ≤ 0.028) and ISDA (all *r* ≤ 0.688, *p* ≤ 0.018) scores. As such, age was included as a covariate for all Pearson R correlations evaluating these measures. Paired sample *t*-test comparisons were used to examine change across the MoCA original and alternate scores, the CVLT-3 performance on trials 1–5 as well as the differences between free and cued delayed recall abilities. All reported statistics reflect two-tailed significance values. Bonferroni corrections were applied when needed.

## Results

Performance on the standard MoCA, including the MoCA delayed recall subtest, did not relate with performance on the immediate or delayed recall subtests of the CVLT-3, regardless of MoCA scoring method used. CVLT-3 subtest performance scores thought to reflect encoding abilities correlated with the ISDA encoding index, with the exception of learning slope. The ISDA retrieval index was not associated with any CVLT-3 measures; however, the alternate ISDA retrieval scoring method was related to the CVLT-3 long-delay free recall subtest. Consolidation abilities, which are only distinctly defined by the ISDA, were associated with CVLT-3 immediate recall trials 2 and 3 as well as both short and long delay cued recall subtests and recognition-hits, but not the delay free recall subtests.

### Discrete Descriptive Statistics of Cognitive Tests

#### Montreal Cognitive Assessment

Similar to Parada et al. (2020), CI users showed the largest change in passing rate when the delayed recall subtest of the MoCA was removed (i.e., significant differences in passing rate between a.) the original with alternate 2 and 3 and, b.) alternate 1 with alternate 2 and 3 MoCA scoring methods; all *p* ≤ 0.039 and *p* ≤ 0.006, respectively). This was not observed between the original and alternate 1 (*p* = 0.250) or between alternate 2 and 3 scoring methods (*p* ≥ 0.999; see Table 5 for the means of each scoring method and passing rate).

**TABLE 5:** MoCA participant scores across the different scoring methods.

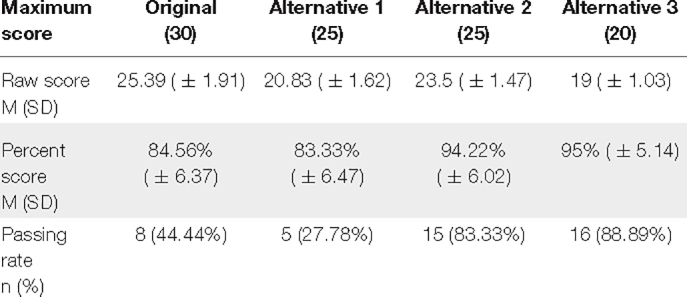

#### California Verbal Learning Test, Third Edition

**FIGURE 2:**
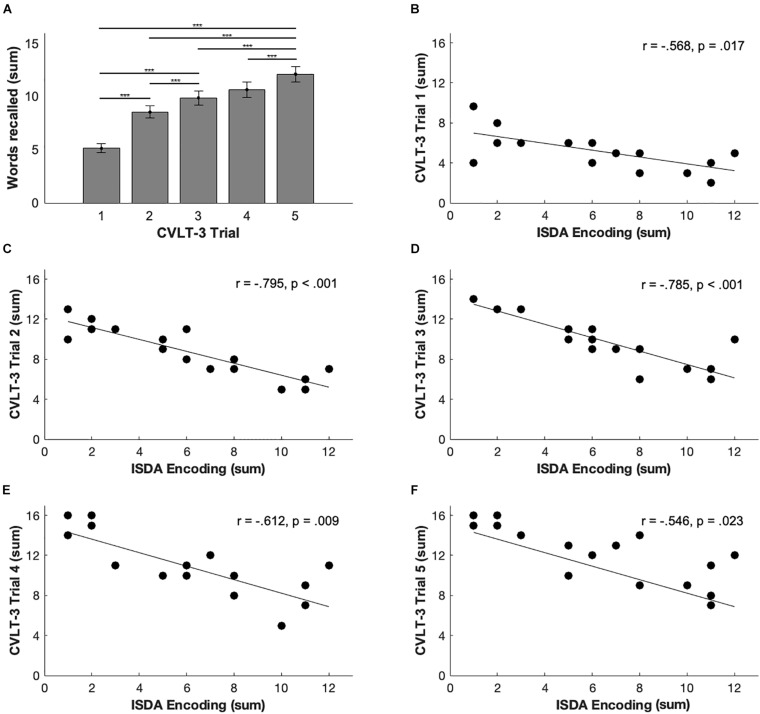
CVLT-3 and ISDA relationships. **(A)** Means and SDs for all participants across trials 1–5. Significant differences were noted between all trials except for trials 3 and 4 with Bonferroni correction *** ≤ 0.001. **(B–F)** Correlation plots indicating the relationship between each trial and the ISDA encoding measure. Significant correlation between each of the trials 1–4 with the ISDA measures except for trial 5 which did not meet significance with Bonferroni correction; *α* = 0.017.

Paired sample *t*-tests between immediate recall trials 1–5 demonstrated significant differences between all trials [see Figure 2; all *t*(17) ≤ −4.44, *p* ≤ 0.001; Bonferroni adjusted *α* = 0.005] with the exception of trials 3 and 4 not being significantly different from each other [*t*(17) = −2.23, *p* = 0.039]. Additionally, paired samples *t*-tests between delayed recall subtests demonstrated a significant difference in performance for scores on the short-delay free recall subtest (*M* = 10.06, *SD* = 3.99) and short-delay cued recall subtest (*M* = 11.28, *SD* = 3.04) [*t*(17) = −3.05, *p* = 0.007] and a marginally significant difference in performance for the long-delay free recall subtest (*M* = 10.33, *SD* = 4.09) and long-delay cued recall subtest [*M* = 11.33, *SD* = 3.43; *t*(17) = −2.34, *p* = 0.032].

#### Item-Specific Deficit Approach

On average, participants received the highest ISDA scores (and therefore experienced the largest deficit) on the encoding index (*M* = 6.38, *SD* = 3.65), followed by retrieval (*M* = 4.28, *SD* = 2.72), and consolidation (*M* = 2.78, *SD* = 2.56).

### Analysis of Montreal Cognitive Assessment Delayed Recall Performance in Relation to California Verbal Learning Test, Third Edition Performance

Incongruent with our initial predictions, no relationships were observed between MoCA performance (for any scoring method) and CVLT-3 performance (all *ρ* ≤ 0.410, *p* ≤ 0.023; Bonferroni adjusted *α* = 0.013) or ISDA indices (all *r* ≤ 0.103, *p* ≤ 0.797). MoCA delayed recall subtest scores did not relate to CVLT-3 performance or calculated ISDA indices (all *ρ* ≤ 0.369, *p* ≤ 0.132).

### Relationships Between California Verbal Learning Test, Third Edition Performance and Item-Specific Deficit Approach Indices

To understand the relationship between these two scoring methods in this patient population, correlational analyses were performed. A Bonferroni correction of *α* = 0.017 was applied to all correlations between CVLT-3 and ISDA scores. In accordance with our predictions concerning encoding and immediate free recall, performance on all CVLT-3 immediate recall measures related to the ISDA encoding index (all *r* ≤ −0.568, *p* ≤ 0.017) with the exception of trial 5 (*r* = 0.546, *p* = 0.023; see Figure 2). Performance on CVLT-3 immediate recall trials 2 and 3 were related to the ISDA consolidation index (all *r* ≤ −0.596, *p* ≤ 0.013). None of the immediate recall measures were related to the ISDA retrieval index (all *r* ≤ 0.116, *p* ≤ 0.987).

CVLT-3 short-delay free recall and short-delay cued recall measures were related to the ISDA encoding index (all *r* ≤ −0.546, *p* ≤ 0.009). Performance on both CVLT-3 cued delay recall subtests (short and long) and recognition-hits were related to the ISDA consolidation index (all *r* ≤ −0.580, *p* ≤ 0.015). While we expected delayed recall measures to correlate with ISDA retrieval scores, none of the CVLT-3 subtests were related to the ISDA retrieval index (*r* ≤ 0.116, *p* ≤ 0.987), but the CVLT-3 long-delay free recall subtest did relate to the alternate ISDA retrieval index (*r* = 0.641, *p* = 0.006). No other relationships between the alternate ISDA retrieval index and the CVLT-3 scores were observed (all *r* ≤ 0.149, *p* ≤ 0.696; see Table 6).

**TABLE 6:** Pearson correlations between the CVLT-3 and ISDA.

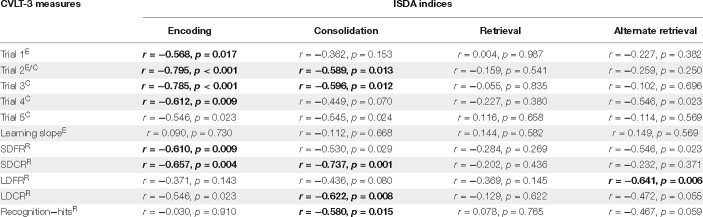

*SDFR, short delay free recall; SDCR, short delay cued recall; LDFR, long delay free recall; LDCR, long delay cued recall; E, encoding measure; C, consolidation measure; R, retrieval measure.*

*Bolded, significant with Bonferroni correction.*

## Discussion

The primary aim of this study was to further examine delayed recall performance in experienced, high-performing CI users to better understand the underlying memory processes characteristic of this group. The CVLT-3 was employed as a comprehensive test of delayed recall, in contrast with the MoCA, which includes only one delayed recall subtest. Incongruent with our predictions, performance on the MoCA delayed recall subtest did not relate to performance on any CVLT-3 subtests. While the CVLT-3 and MoCA are both widely used by clinicians, the lack of relationship between delayed recall performance as measured by the two tests highlights their differences when applied to CI users.

To better understand the lack of relationship between MoCA and CVLT-3 performance observed here, the differences between two tests need to be explored. Delayed recall consists of 45.5% of the overall score of the CVLT-3, whereas it only accounts for 16.6% of the overall score of the MoCA. Additionally, the delayed recall periods are different, with the CVLT-3 instructing for an approximately 20 min delay period between short-delay cued recall and long-delay free recall subtests, and the MoCA instructing for a 5 min delay period between initial immediate recall memory and delayed recall subtests (see Table 2). These differing delay periods have the potential to allow for different levels of consolidation and forgetting of stimuli based on the capacity of each memory system. This is because the assumed storage capability of STM is about 30 s with a capacity of about seven numeric digits (Miller, 1956; Craik and Lockhart, 1972). The longer delay period in the CVLT-3 may result in greater forgetting of target stimuli (i.e., the 16 words the participant is attempting to remember). Conversely, the word list in the CVLT-3 is read to the participant five times, allowing for further consolidation of the stimuli through repetition, while the MoCA word list is only read twice. Additionally, the CVLT-3 includes scoring for delay cued recall, whereas the 7.1-3 versions of the MoCA do not. The inclusion of delay *cued* recall, which utilizes semantic categories for recalling stimuli, allows for the exploration of other types of delayed recall that may use different cognitive processes from delay *free* recall. Taken together, differences in scoring methodology, delay periods, repetition and cueing complicate comparing delayed recall performance measured of the MoCA v. the CVLT-3.

Previous literature has examined cognitive performance in relation to hearing abilities for the MoCA (Dupuis et al., 2015; Ambert-Dahan et al., 2017; Lim and Loo, 2018; Hillyer et al., 2020; Parada et al., 2020; Shen et al., 2020; Utoomprurkporn et al., 2020) and CVLT-3 (Kramer et al., 2018; Moseley, 2018; Pisoni et al., 2018; Chandramouli et al., 2019), demonstrating that differences in cognitive abilities due to sensory impairments like HL should be taken into account during test administration. Our current findings present the first application of the ISDA to a population with HL. One potential strength of the ISDA is the ability to counteract the effects of inattention by measuring performance at the item level rather than by overall trial performance (Wiegner and Donders, 1999; Wright et al., 2009). This item-level approach may also offer the ability to account for the fact that not all 16 words are always properly encoded by tabulating scores based only on the words successfully recalled. The original CVLT-3 scoring method tabulates each score as a proportion of all 16 words, whether all 16 words were encoded at some point or not. In this study, our participants repeated 91% or approximately 15 of the 16 target words at least once across the five immediate recall trials, whereas they repeated 78% or approximately 13 out of the 16 target words at least once across all delayed recall subtests. These results support similar findings by Parada et al. (2020) suggesting that poorer delayed recall performance may be a result of an impaired consolidation and/or retrieval mechanism than an inability to properly hear and encode the words during the initial immediate recall trials. While the majority of words were properly encoded in our participant population, individuals with poorer speech discrimination could benefit from this item-level approach, as it may mitigate failures to encode test stimuli due not hearing or mishearing. More research is needed to address this.

In line with our secondary aim to relate ISDA indices with equivalent CVLT-3 subtests, we found that performance on the CVLT-3 immediate recall measures thought to reflect encoding abilities were related to the ISDA encoding index, whereas consolidation measures demonstrated a different pattern. CVLT-3 cued delay recall performance related to the ISDA consolidation index, but CVLT-3 free delay recall performance did not. These results may be driven by the ISDA scoring criteria for consolidation, which requires participants to recall a target word on at least one of the four delayed recall subtests in order to *not* receive a point toward their consolidation index score. In other words, the inability to recall the target word on any delayed recall subtests (i.e., score of 0 out of 4) would result in an increased consolidation deficit. Consequently, it stands to reason that the ISDA consolidation index may relate to performance on delayed recall subtests where stimuli were remembered most frequently, which in our study were the delay cued subtests. Previous research in individuals with normal hearing has demonstrated that the cognitive processes associated with cued and free recall are different (Nobel and Shiffrin, 2001; Brainerd et al., 2002; Nyberg et al., 2002; Padilla-Walker and Poole, 2002; Ivanoiu et al., 2005; Cerciello et al., 2017). Similarly, in CI users, a potential difference in the cognitive processes associated with cued recall could be that cued recall, much like a recognition task (e.g., recognizing target words among other distractor words), is both a measure of familiarity and recall (Bastin and Van der Linden, 2003). Studies involving recognition tasks have indeed suggested that familiarity and recall are processes occurring independently of one another in the brain (Aggleton and Brown, 2006). In the context of the CVLT-3, the categorical cues given during the delay cued recall subtests may tap into a pre-existing system of familiar words already existing in the participant’s memory. With regards to our study, an increase in performance was observed when semantic cues were provided (1 extra word on average recalled; range of 0–7 words). Participants who may have used the provided CVLT-3 semantic cues to recall more words demonstrated proper encoding; however, they may have had more difficulty with retrieval since the benefit of semantic cues has been shown to demonstrate a retrieval deficit (Farrer and Drozdick, 2020). Participants who did not demonstrate an increase in performance (and therefore did not benefit from provided CVLT-3 cues) may have already been using their own semantic strategies to recall words, such as constructing unique semantic categories or using rehearsal devices. In future administrations of the CVLT-3, asking participants whether they constructed their own semantic cues or not prior to providing the CVLT-3 semantic cues would offer additional insight.

It would be expected that CVLT-3 retrieval performance be related to the ISDA retrieval index; however, in our study, we found that ISDA retrieval deficit calculated with the original scoring method did not relate to performance on any CVLT-3 retrieval measures. Alternately, our less stringent scoring method to calculate the ISDA retrieval deficit did reveal this expected relationship. This alternate ISDA retrieval index was calculated with relaxed criteria; specifically, a participant could recall a word across three or four subtests and avoid increasing their overall retrieval deficit score. In other words, a participant’s retrieval deficit score was not increased if they failed to recall a word on one subtest, but rather, they were given a point toward their retrieval deficit score if they failed to recall a word for two or more subtests. This alternate index still required the participant to recall a target word on at least one short delay and long delay condition. Our data indicated that 8.33 words on average satisfied the original ISDA retrieval index criteria and were recalled across all four delayed recall subtests, whereas with the less restrictive retrieval index, 10.44 words were recalled on at least three or more delayed recall subtests. While the mean difference between the original and alternate retrieval indices is only two words, the less restrictive retrieval index significantly related with a CVLT-3 retrieval measure (long-delay free recall). Our results highlight that the alternate ISDA retrieval index may be beneficial in capturing an element of retrieval that is not part of the ISDA original score. Indeed, long-delay free recall is often considered to be a more pure measure of retrieval abilities based on its lack of interference from distractor words (i.e., list B; Ebert and Anderson, 2009; Farrer and Drozdick, 2020). To understand the relationship between our alternate retrieval measure and the CVLT-3 short and long delay delayed recall conditions, we calculated which of the four conditions a participant was most likely to forget a target word. We found that when a word was recalled three out of the four possible times that the condition where a word was most likely forgotten was long-delay free recall. Specifically, we determined that target words were forgotten 21, 11, 27, and 14 times across all participants on the CVLT-3 short-delay free, short-delay cued, long-delay free, and long-delay cued subtests, respectively. With the original ISDA scoring method, these forgotten words would count toward an overall retrieval deficit without accounting for the fact that the majority of these instances of forgetting occurred during the long-delay free recall subtest. Perhaps our less stringent ISDA retrieval scoring method provided more sensitivity to the presence (or absence) of a retrieval deficit and therefore revealed this expected significant relationship with long-delay free recall ability. However, additional work is needed to determine the clinical utility of this alternate score in a CI patient population as well as expanding this scoring method to a normal hearing population.

### Limitations and Future Work

This study had several limitations, mainly in its relatively small and specific sample size, and lack of age-matched normal hearing control subjects. Our study consisted of mostly older adults with high-performing speech perception abilities and thus, our findings may not generalize to all CI users. Future research should examine delayed recall abilities and apply the ISDA to other groups of CI users such as those with lower speech perception performance (Moberly et al., 2016), single sided deafness (SSD; Sharma et al., 2016), and younger participants (Cartocci et al., 2019) to further explore the validity of these constructs. Although our sample size is not atypical of research surrounding CIs (Moberly et al., 2018; Sladen et al., 2018; Mancini et al., 2020; Zhan et al., 2020), this may have limited our ability to detect smaller differences in performance and may have contributed to a lack of relationship between the MoCA and CVLT-3 measures. While the ISDA has been used in other clinical populations (Wright et al., 2009, 2010; Cattie et al., 2012; Oltra-Cucarella et al., 2014; Tayim et al., 2016; Basso et al., 2021), this was the first study to apply the ISDA scoring method to a CI population, and thus this study offers an additional set of constructs, rarely used in previous CI studies, to describe delayed recall abilities in this population. Another limitation of this study was that the modality of test presentation was either auditory (CVLT-3) or auditory-visual (MoCA), which introduces additional difficulties for individuals with HL. We previously explored differences in modality with the MoCA and a version of the MoCA for hearing-impaired populations (i.e., the HI-MoCA, a version of the MoCA that is presented entirely in the visual modality via PowerPoint presentation) that demonstrated little influence on overall performance for CI users (Lin et al., 2017; Parada et al., 2020). While previous literature has explored the utility of a non-auditory CVLT-II (Pisoni et al., 2018), future research could examine specific cognitive relationships alongside speech perception performance with a non-auditory administration of the CVLT-3. Additionally, employing tests such as the Free and Cued Selective Reminding Test (FCSRT; Buschke, 1984), which alternative to the CVLT-3 provides category cues to participants at the beginning of the assessment, could help further elucidate differences between free and cued delayed recall abilities in this population.

## Conclusion

While CVLT-3 and ISDA measures did not relate with the MoCA, our work indicates that the ISDA can successfully be applied to CI users to quantify delayed recall ability. Specifically, the advantage of the ISDA is that it provides a discrete measure of consolidation, although our results also highlight that an alternate ISDA retrieval score may be needed. Our work, however, should be considered preliminary as additional work is needed to assess the clinical utility of the original and alternate ISDA scoring methods in both a normal hearing and more expansive CI patient population.

## Data Availability Statement

The datasets presented in this article are not readily available because the Auditory Research Laboratory is part of a hospital system that does not allow for data sharing due to patient privacy requirements. Requests to access the datasets should be directed to corresponding author.

## Ethics Statement

The studies involving human participants were reviewed and approved by the Swedish Medical Center Institutional Review Board. The patients/participants provided their written informed consent to participate in this study.

## Author Contributions

NB, JP and AP-C completed the statistical analysis of data. NB created the figures and tables. NB, EE and AP-C wrote the manuscript. JP and JH contributed to editing the manuscript. All authors approved the submitted version, contributed to the study design, and participated in data collection.

## Conflict of Interest

The authors declare that the research was conducted in the absence of any commercial or financial relationships that could be construed as a potential conflict of interest.

## Publisher’s Note

All claims expressed in this article are solely those of the authors and do not necessarily represent those of their affiliated organizations, or those of the publisher, the editors and the reviewers. Any product that may be evaluated in this article, or claim that may be made by its manufacturer, is not guaranteed or endorsed by the publisher.
